# How leaders in mental health services shape workforce training outcomes: goals, actions, and mechanisms of change

**DOI:** 10.3389/frhs.2026.1784462

**Published:** 2026-04-13

**Authors:** Emma Högberg Ragnarsson, Terese Stenfors, Lise Bergman Nordgren, Clara Hellner, Tobias Lundgren, Sara Ingvarsson

**Affiliations:** 1 Centre for Psychiatry Research, Department of Clinical Neuroscience, Karolinska Institutet, and Stockholm Health Care Services, Stockholm, Sweden; 2 Department of Learning, Informatics, Management and Ethics, Karolinska Institutet, Stockholm, Sweden; 3 Division of Psychiatry, Örebro, Sweden; 4 Department of Medicine, Faculty of Medicine and Health, Örebro University, Örebro, Sweden

**Keywords:** implementation strategies, manager perspectives, mechanisms of change, mental health services, operant learning theory, qualitative content analysis, work environment, workforce training

## Abstract

**Objective:**

Workforce training is a widely used implementation strategy to improve care within mental health services, yet evidence for sustained changes in clinical practice remains inconsistent. Managerial actions are likely critical for translating training into practice, but little is known about how leaders conceptualize training objectives or which mechanisms of change they rely on. This study examined which outcomes mental health managers value from clinical staff training and the strategies they use to support effectiveness.

**Method:**

In-depth interviews were performed with 14 managers from eight Swedish regions, and data were analyzed using two forms of qualitative content analysis: Training objectives were inductively coded into categories, while reported strategies were deductively organized using an operant learning framework to identify behavior-change mechanisms.

**Results:**

Managers described training as serving multiple purposes organized in two overarching aims: *Care Delivery Goals*, including clinical impact and service provision, and *Work Environment Goals*, encompassing staff wellbeing and connectedness among colleagues. Reported strategies aligned with the four predefined categories—Antecedents, Monitoring, Consequences, and Processes & Resources—and one additional category developed during coding: Preparatory strategies. Across strategies, mechanisms essential for sustaining new skills, such as task clarification, outcome monitoring, and performance-contingent feedback, were inconsistently applied.

**Conclusions:**

This study demonstrates that leaders attribute a wider range of purposes to workforce training than is typically assumed in training and implementation research, encompassing not only clinical or competence-related outcomes but also central work environment and organizational goals. An operant analysis of their strategies clarifies the change mechanisms managers rely on and highlights the importance of intentional, strategic-level actions—including preparatory strategies that shape who is trained, in what, and why. Furthermore, the findings inform practical recommendations that emphasize linking training initiatives to organizational priorities and systematically employing strategies such as monitoring, feedback, and task clarification. Integrating these elements can strengthen the design, implementation, and long-term impact of staff training, ultimately supporting better patient outcomes, improved staff wellbeing, and more effective use of limited resources.

## 1 Introduction

Clear training objectives are critical for optimizing learning outcomes, as they provide organizations, trainers, and trainees with a shared understanding of the expected change resulting from staff training (1–3). While intended learning outcomes (ILOs) are typically articulated within individual clinical training courses, organizations often lack explicit expectations for training objectives and learning transfer [i.e., applying the new knowledge and skills into the workplace (4, 5)]. Given the large expenditure on training within mental health services (6), this ambiguity may result in inefficient use of resources and limited return of investment for the organization. However, little is known about what training objectives are perceived as meaningful from a mental health organizational perspective, or how leaders in mental health services experience their role in achieving these goals.

The effectiveness of continuing education and training initiatives for mental health practitioners is a subject of ongoing scholarly and practical interest. In clinician training research, training is often operationalized as structured actions with the objective of increasing sustained knowledge or performance. Noe and Schmitt [(7), pp 497] states: “Training can be defined as a planned learning experience designed to bring about permanent change in an individual's knowledge, attitudes, or skills”. In line with this definition, research has examined the effects of therapist training. For example, a recent meta-analysis by Ragnarsson et al. (7), showed that therapist training can produce short-term changes in clinician behavioral outcomes, such as competence and adherence. However, other studies show that achieved behavioral training outcomes may be diminished six months after training, if no additional support is added (8). Furthermore, it has been suggested that over time, therapists tend to become less adherent to treatment protocols they have been trained in (9, 10).

Other researchers conceptualize training as an implementation strategy, highlighting clinical outcomes as the ultimate goal of training (11, 12), with mixed support for effectiveness. For example, Grey and colleagues (13) concluded that a brief cognitive-behavioral therapy (CBT) training improved patient outcomes, whereas Bein and colleagues (14), in a similar study, failed to find an effect of training on patient outcomes.

The scarce evidence for sustained or distal effects has prompted increased attention to the “survival” or transfer of training outcomes in real-world settings, by studying conditions for optimal learning transfer (15, 16). Furthermore, considering the substantial investment that mental health services allocate to staff training (6), inconsistent effects pose challenges for organizations to get return on investment, highlighting the need to better understand the conditions under which training translates into sustained practice change. Prior research points to employees’ opportunities to perform newly learned skills, organizational climate, and leadership behaviors as critical contextual determinants of successful learning transfer and implementation (5, 17). Together, this suggests that while training itself may produce short-term outcomes, the organizational environment—and leader's actions in particular—is critical for maintaining new practices over time and getting the most out of workforce training efforts.

To better understand the mechanisms involved in this process, a theoretical framework is needed. Operant learning theory (18, 19), including its applications in applied behavior analysis (20) and Organizational Behavior Management [OBM (21)], offers a functional, evidence-based approach for analyzing how environmental contingencies shape behavior. Within this framework, leadership strategies such as task clarification, reminders, monitoring, feedback, and reinforcement can be understood as mechanisms that support or hinder the development, adoption and sustained use of knowledge and skills (22). This functional emphasis enables the identification of transferable interventions, offering utility across different organizational contexts and workforces (i.e., as opposed to lists of discrete, context-specific strategies).

Nonetheless, without a shared understanding of the intended outcomes of training in mental health services, meaningful evaluation of its effectiveness remains limited. Despite the central role leaders play in shaping the conditions for sustained training outcomes, little is known about how they conceptualize the objectives of clinical staff training or which strategies they employ to support its impact. To address this gap, the current study investigates:

1. What objectives of clinical staff training do leaders in Swedish mental health services describe?
2. What strategies do leaders employ to ensure that clinical staff training fulfills these objectives, and how can the change mechanisms of these strategies be understood using an operant learning theory framework?

## 2 Materials and methods

Given the lack of prior research, we adopted an exploratory approach based on individual, in-depth interviews with mental health managers and qualitative content analysis. Researcher positionality and reflexivity are detailed in Appendix 1.

### 2.1 Setting and recruitment

Swedish mental health services are part of the publicly funded, de-centralized healthcare system, which is organized and administered by 21 counties across the country. In line with regulations issued by the Swedish Work Environment Authority, managers are responsible for ensuring a safe and healthy work environment (23). Furthermore, according to union agreements with the health care providers, each employee is entitled to annual performance reviews as well as an established competence development plan (24, 25).

The study was conducted within mental health services in eight different counties across the country. Inclusion criteria were managers at any level who are actively involved in staff-training decisions, either in their capacity as line managers and/or through a specific assignment or role. Purposive sampling was employed via e-mail. We started by contacting a national network for managers in mental health services, sharing information about the study and a registration link. Since organizational conditions were expected to vary between rural and metropolitan settings concerning e.g., labor-market, workforce supply, and access to training programs, we strived for both variation and representation of geographical location to achieve richness and breadth. To this end, complementary e-mail recruitment was directed toward a metropolitan area to balance the initial rural representation in the sample.

### 2.2 Ethical considerations

The study was reviewed by the Swedish Ethical Review Authority and deemed not to require formal ethical approval (dnr: 2024-00245-01). All participants received written information about the study and gave informed consent both in writing before participation and verbally before the start of each interview. In line with GDPR and ethical research standards, transcripts were pseudonymized, and all data were handled confidentially. Given the small and identifiable population of mental health service managers in Sweden, we report only group-level characteristics to ensure that individual participants cannot be identified in the reporting of results.

### 2.3 Participants

We estimated that 10–15 participants would yield strong information power, based on usage of narrow research questions, dense sample specificity, a strong quality of dialogue and applying an established theory (26, 27).

In total, 14 participants meeting the inclusion criteria were interviewed between Oct. 2024 and Feb. 2025. All except one were mental health professionals by training. For more information, see Table 1.

**Table 1:** Summary of participant characteristics.

Characteristic	*N* = 14
Gender	
Female	7 (50%)
Male	7 (50%)
Age, mean (range)	52 (38–65)
Region	
Major metropolitan	7 (50%)
Rural/non-major metropolitan	7 (50%)
Type of mental health service	
Adult oriented	9 (64%)
Child and adolescent oriented	5 (36%)

### 2.4 Data collection

We developed a semi-structured interview guide with open-ended questions, to balance a systematic approach with the flexibility to explore interesting topics (28). Interviewee statements that related to the second research question, i.e., concerning strategies to achieve intended objectives, were followed by targeted prompts to collect detailed descriptions of the strategies mentioned (29). The guide was piloted before the study began, which contributed to revisions of the guide. Data from the pilot were not included in the analysis. The interview guide is provided in the Supplementary Material S1.

Two members of the research team (EHR, SI) conducted the interviews one-on-one, listened to each other's interviews, and kept an ongoing dialogue between them on any issues or needs for updates of the guide. To include participants from all parts of Sweden, interviews were conducted via video conference calls (i.e., Zoom), and audiotaped. Interviews lasted between 38 and 52 min.

### 2.5 Data analysis

Two complementary analytic strategies were applied to address the research questions. To answer research question one—what objectives of clinical staff training managers describe—we employed conventional content analysis to inductively identify codes and categories (29). This approach was chosen to explore participants’ experiences without imposing preconceived categories. During the analysis, we strived to stay close to the data by using low-interpretation, low-abstraction categorizations (28, 30). For research question two—what strategies managers use to fulfill these objectives—directed content analysis was applied (29). To this end, we developed a codebook based on operant learning theory to guide the analytic process.

Interviews were automatically transcribed using Microsoft Word's transcription feature. To ensure both accuracy and familiarity with the material, the first author proofread and pseudonymized all transcripts. The finalized transcripts were then imported into NVivo 14 (31) for further analysis.

For further details regarding study quality and reporting, the COREQ checklist (32) is provided in the Supplementary Material S2.

### 2.6 Coding and categorizing

Coding was conducted by EHR with ongoing discussions and peer debriefings with SI. To enhance analytical rigor, keep an ongoing reflective dialogue, and ensure alignment with the research questions, the full research team convened on several occasions to review and refine the coding framework collaboratively.

During initial data preparation we identified meaning units, which consisted of one or a few sentences, and coded them inductively (30). All codes were initially sorted based on relevance to one or both research questions.

For research question one, relating to training objectives, manifest content was structured into codes and iteratively grouped into categories and subcategories at a descriptive level (28, 30). These categories were not predefined but developed through close engagement with the data.

For research question two, relating to strategies, we developed a codebook based on operant learning literature related to management, employee development, and staff performance. This was achieved by combining knowledge on effective management by Komaki (33) showing the importance of monitoring and consequences for effective leadership, with the performance assessment framework Performance Diagnostic Checklist is designed to assess variables that impact employee performance (PDC; 21, 35, 36). From the data, we identified and coded meaning units where managers described actions intended to reach a previously stated value of training, i.e., the strategy and its intended function, and organized these using the codebook. During coding, we noticed frequent use of strategies that did not align with the operant framework, as they reflected managerial activities undertaken to prepare for training initiatives rather than aiming to influence employee behavior. Hence, we formed an additional category—preparatory strategies—to capture these efforts. Detailed definitions of all categories follow below.

#### 2.6.1 Antecedents

Antecedents are conceptualized as strategies where a manager “instructs, reminds, or conveys an expectation of performance” to the employee [(34), p. 39]. In the three-term, or ABC, contingency, antecedents (A) occur before a behavior (B) and signals what consequence (C) is expected. The employee behavior, or task, in question can take place before training (e.g., apply to a course), during training (e.g., take notes, participate in all learning activities), or after training (e.g., perform new assignments). By using effective antecedents (A), a manager can increase the likelihood of a desired employee behavior (B).

#### 2.6.2 Monitoring

Monitoring referes to collecting information about training-related performance (33). This can be done in using direct observation, indirect observation, self-monitoring, and collecting information from other sources, e.g., patient outcome measures, incidents reports, employee satisfaction surveys.

#### 2.6.3 Consequences

“Consequences communicate an evaluation of or indicates knowledge of another's performance, where the indication can range from highly evaluative to neutral” [(34), p 38] and is. In terms of the ABC contingency, the likelihood for a future behavior (B) is influenced by its consequences (C), either by increasing or decreasing the likelihood. Examples of consequences are feedback, the effort it takes to complete a task (i.e., perform the behavior), and whether the task is possible to prioritize over other tasks.

#### 2.6.4 Processes & resources

Processes & resources are conceptualized as environmental variables that influence the probability of behavior but do not function as direct contingencies (i.e., not immediate antecedents or consequences). They are critical in determining whether desired performance is likely or even possible, and therefore integrated into behavior-analytic organizational assessment tools such as the PDC (20). Examples include the availability of time and materials, scheduling, and appropriateness of workflows.

#### 2.6.5 Preparatory strategies

Preparatory strategies reflect managerial activities utilized before training, such as selecting which employees who both needed and were suitable to attend training, determining which training programs to prioritize, or ensuring that considered training programs met certain quality standards.

## 3 Results

### 3.1 Objectives of clinical staff training—research question one

**Figure 1:**
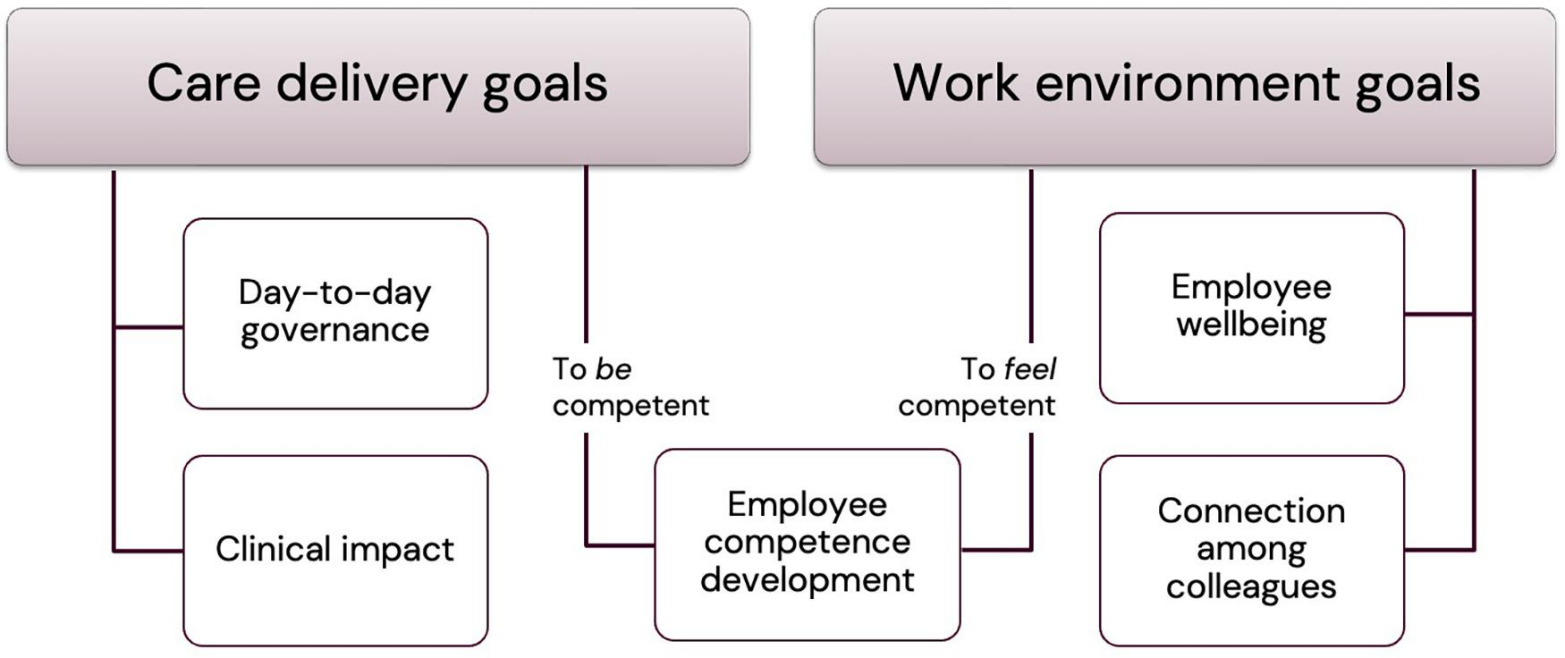
Goals of staff training. The top bars depict main categories. Boxes contain subcategories. The middle box represents the fifth subcategory, with bearings on both main categories.

Two main categories, and five subcategories, of training objectives were identified: *Care Delivery Goals*, consisting of (1) day-to-day governance and (2) clinical impact; and *Work Environment Goals*, consisting of (3) employee wellbeing and (4) connection between colleagues. A fifth sub-category, (5) employee competence, was identified, relating to both main categories depending on whether “being competent” or “feeling competent” was emphasized. Categories and subcategories are depicted in Figure 1 and detailed below. A coding tree with example quotes is provided in Appendix 2.

#### 3.1.1 Care delivery goals

This first main category refers to staff training outcomes with direct implications for organizational excellence, i.e., delivery of psychiatric care in alignment with the organization's mandate. Two subcategories were identified: Day-to-day governance, and Clinical impact.

##### 3.1.1.1 Day-to-day governance

Day-to-day governance encompasses objectives related to the prerequisites for the effective functioning of daily operations, such as adequate staffing, effective communication, and the establishment of standardized work processes across the organization. One interviewee expressed that assembling staff during training initiatives foster similar work processes across the organizations, both through learning the same methods, but also because it provides an opportunity to compare and discuss different practices applied in the units. Interviewees also mentioned training as a way to keep and attract staff, even when the training is not immediately applicable in their unit.

##### 3.1.1.2 Clinical impact

Clinical impact refers to the delivery of high-quality care to patients in alignment with clinical guidelines, while maintaining safety for both patients and staff. The interviewees described that being able to deliver specific methods that were prescribed in local care processes or national guidelines was an essential goal of training efforts. Others emphasized the need to be prepared for upcoming patient needs or announced changes to clinical guidelines. Some respondents utilized staff training as a tool to address safety issues, either actual or anticipated. Respondent 4 described advocating for this need: *“*I told decision-makers, ‘If we don't invest in competence, maybe we should stop upgrading our medical equipment too’. Because to me, staff expertise is just as critical for patient safety.”

#### 3.1.2 Work environment goals

The second main category relates to staff training outcomes with implied but indirect bearings on organizational outcomes, concerning topics like employee motivation and organizational climate, rather than objective employee performance or behavior. The two subcategories identified were Employee wellbeing, and Connection among colleagues.

##### 3.1.2.1 Employee wellbeing

Employee wellbeing refers to training as a way to reward staff, reduce stress, improve resilience, and indulge in interests. Respondent 10 describes training as possibility to recharge: “I also think it's a nice break from the everyday routine. (…) I think that's what helps you manage and carry on.”

##### 3.1.2.2 Connection among colleagues

Connection among colleagues emphasizes the positive interpersonal aspects that may results from staff training, like informal learning, feeling less alone, and building resilient teams.

##### 3.1.2.3 Employee competence development

This subcategory bridges the two main categories, i.e., Care delivery goals and Work environment goals. Here, continuous competence development is referred to as beneficial *per se*, yet with impact on both clinical work and employee wellbeing implied. As such, this topic describes proximal employee-level outcomes and highlight professional development. One aspect of this subcategory covers employees keeping and increasing their competence, e.g., by staying updated with what is new in the field, and rehearsing or repeating what is already known. Some mentioned how training can foster self-monitoring, and others and how training can impose a long term at-work maturation, all relating to sustained or improved competence over time. The other aspect of *Employee competence development* that was articulated by the interviewees, was the emphasis of its relation to wellbeing-factors, such as resilience and a sustainable work environment. Here, respondents described the importance of employees’ own perceptions of competence and growth.

### 3.2 Managerial strategies analyzed within the operant learning theory framework—research question two

**Figure 2:**
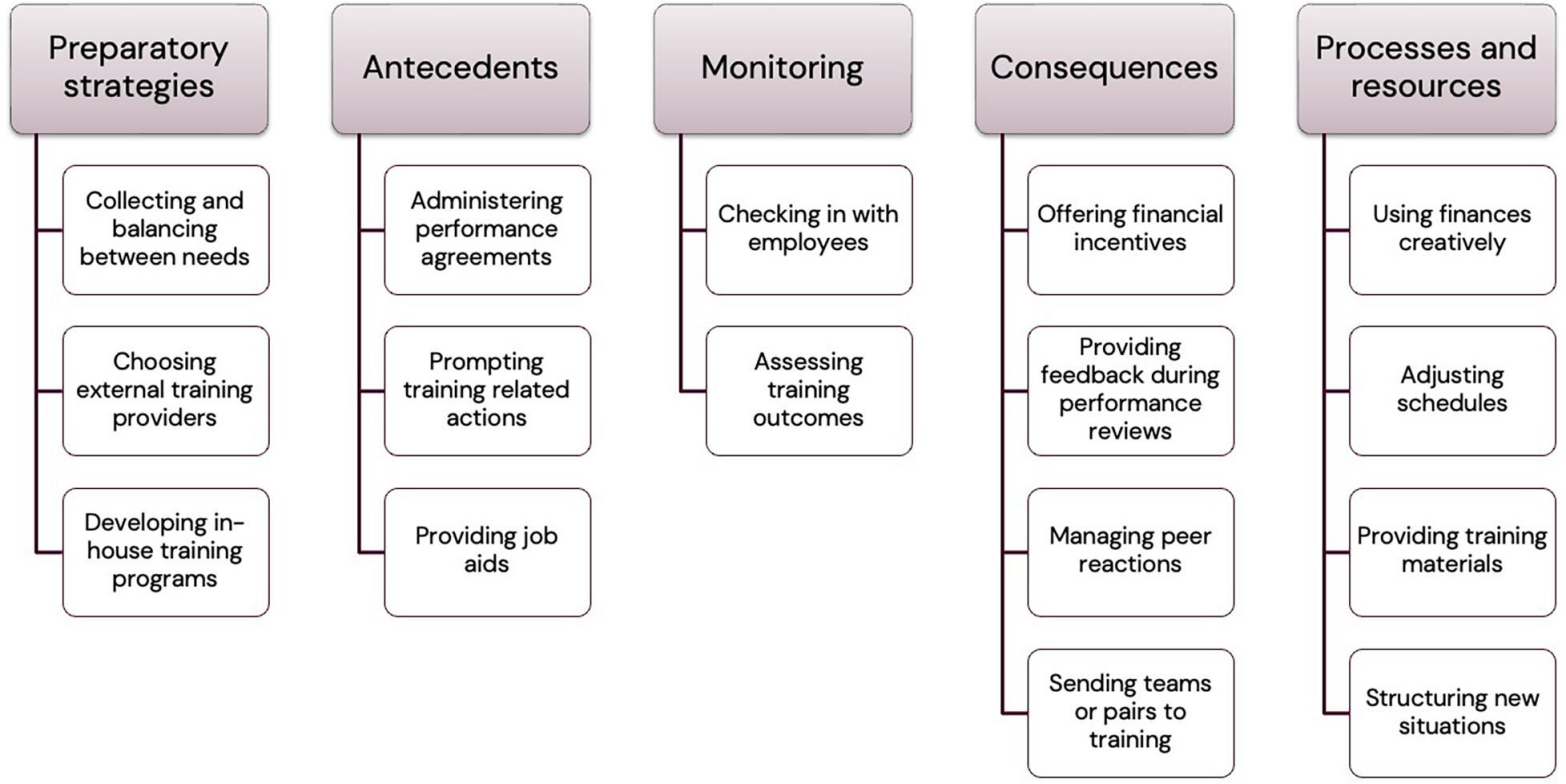
Categories and subcategories of strategies. Top bars depict main categories. Boxes contain subcategories.

Interview data were organized into the five main categories operationalized in the codebook: Preparatory strategies; Antecedents; Monitoring; Consequences; and Processes & resources. (Respondent 12). An overview of the categories and subcategories are provided in Figure 2.

#### 3.2.1 Preparatory strategies

Interviewees reported performing several preparatory strategies that preceded and shaped training implementation. These activities reflected intentional, strategic-level decision-making aimed at aligning training investments with organizational priorities, workforce capacity, and staff needs, thereby guiding the selection of the right employee for the right training at the right time. Three subcategories of such strategies were identified: Collecting and balancing between needs; Choosing external training providers; and Developing in-house training programs.

##### 3.2.1.1 Collecting and balancing between needs

Refer to strategies where managers gather information and utilize it to decide who should attend which training programs. Sources of information ranged from routinely mapping organizational competence gaps to responding ad-hoc to employees’ suggestions. Standardized strategies include surveying first line managers to report units’ needs and using descriptive statistics from the clinics. Interviewees mentioned the yearly or biyearly performance review as a natural place to start, while other highlighted attending clinical meetings or working clinically themselves as important to understanding competence needs. Interviewees also talked about dilemmas and difficult considerations when choosing who to send to training. Respondent 6:

It's often the people who are well-educated and have worked a long time and taken lots of courses—they’re the ones always looking out for new courses. They’re the best at putting themselves forward, and it's a delicate matter. That's the staff group you want to keep, normally. They’re the most valuable, and at the same time you can't send them off to a course every year while nobody else gets to go.

##### 3.2.1.2 Choosing external training providers

Several interviewees described measures taken to investigate availability and quality of training programs. Some scanned newsletters and web pages, reviewed course evaluations, or asked employees and other managers for recommendations.

##### 3.2.1.3 Developing in-house training programs

Some interviewees estimated that available training programs did not have the right length, topic or quality, hence decided to arrange brief trainings themselves. Others described in-house training as useful for addressing urgent requests from teams or professions.

#### 3.2.2 Antecedents

This category encompasses strategies intended to activate employee actions in relation to training and training outcomes. The following subcategories were identified: Administering performance agreements; Prompting training related actions; and Providing job aids.

##### 3.2.2.1 Administering performance agreements

Some interviewees described using written or verbal plans where manager and employee defined expected learning outcomes. Respondent 8:

You create an individual professional development plan. An agreement that after completing the training, you’re supposed to use what you’ve learned in your work at the workplace. Then you must set aside meetings with your manager to really go through things like: okay, which patients can we now take on, how much, what kind of supervision might be needed, and so on.

##### 3.2.2.2 Prompting training related actions

Prompting training related actions refers to managers instructing and encouraging employees to apply to training, finish training, or implement new skills developed during training. “I followed up with the person and, in a way, encouraged them a bit or sort of pressed that, yes, it's important that you also complete the training”. (Respondent 1).

##### 3.2.2.3 Providing job aids

Providing job aid refers to the provision of checklists, treatment manuals or other material that remind and support the employee during at-work implementation of knowledge and skills developed during training. “We provide treatment support for all conditions, and it's very practical—guides, checklists, and concrete tools that help apply the knowledge.” (Respondent 14).

#### 3.2.3 Monitoring

Monitoring refers to managerial initiatives to gather information about how the training and post-training application of skills are going. The two subcategories relating to monitoring strategies—Checking in with employees and Assessing training outcomes—are detailed below.

##### 3.2.3.1 Checking in with employees

Checking in with employees encompasses situations where a manager, planned or spontaneously, e.g., upon meeting the employee in the corridor, asks how training or training outcomes are developing. Respondent 1 provided this example: “During a training, I usually check in, asking, ‘How's the training going? How do you feel about it?” Check-ins were also reported to take place during case conferences, team meetings, multidisciplinary meetings and administrative meetings.

##### 3.2.3.2 Assessing training outcomes

When interviewees were asked if and how they know if stated training objectives are fulfilled, many acknowledged lacking structured strategies to monitor the effects of training. One interviewee suggested drawing on the employee survey to get a sense of how staff feel about their competence. Interviewees talked about outcome monitoring as something they would like to do more of, like Respondent 14:

Right now, we don't have any concrete follow-up on this. However, we often keep it in mind when talking to unit managers—that there should be some knowledge transfer, that it should work in practice, and that the knowledge gained should be applied in everyday work. But it's just words; we haven't followed this up. Even though we have the vision that we should.

#### 3.2.4 Consequences

This category encompasses managers’ efforts to provide reinforcement or evaluative feedback to the employee, in relation to training participation and previously stated training outcomes. The following strategies were identified: Offering financial incentives; Providing feedback during performance reviews; Managing peer reactions; and Sending teams or pairs to training.

##### 3.2.4.1 Offering financial incentives

Some interviewees declared providing a salary increase as a consequence of finishing a longer continuous professional development program. Respondent 11 discussed financial incentives as needed but not enough to keep staff after training: “It's a matter of a few thousand Swedish crowns [a few hundred euros] a month, but it's rarely something that, over time, will correspond to the changed position these employees have in the labor market.”

##### 3.2.4.2 Providing feedback during performance reviews

This subcategory reflects descriptions of using the performance review to convey evaluations of training participation and/or outcomes. While these meetings were mainly mentioned when discussing ways to gather information about training needs, they were sometimes also utilized to provide feedback: “It's one of the mandatory parts of the employee review, of course, but it's probably not enough since there's such a long time between them. It's more just general encouragement. I believe there are definitely more things we could do.” (Respondent 11).

##### 3.2.4.3 Managing peer reactions

One interviewee described that newly trained employees may meet negative comments from colleagues—risking the trained employee's willingness to apply new skills—and the actions taken to mitigate their impact:

If you know you’re going to get a lot of negative comments aimed at you and the training you attended, it's not very motivating to bring it up again. Sometimes people think, ‘Here's this new thing again, but I’m damn well going to do it the way I’ve always done.’ Then it becomes the manager's responsibility to work on changing attitudes or at least explain to the person who's resistant to change, ‘This is the way we do things now.’ (Respondent 3).

##### 3.2.4.4 Sending pairs or teams to training

Some managers had noticed benefits of sending more than one employee at a time to training to reduce task effort, i.e., lessen the burden for each person. Respondent 10 also described the strategy as timesaving: “For example, we had our whole team away at a conference. When they came back, they were like, ‘Okay, now we can think like this,’ and everyone was on board. So, you didn't have to spend four months anchoring the change with the rest of the group.”

#### 3.2.5 Processes & resources

This category encompasses strategies where interviewees tweak existing processes and resources. The four subcategories, detailed below, include: Using finances creatively; Adjusting schedules; Providing training materials; and Structuring new situations.

##### 3.2.5.1 Using finances creatively

During the interview period (Oct. 2024-Feb. 2025) most regions in Sweden were subjected to financial constraints, consequently leaving little room for organizations to prioritize staff training. This was reflected in the interviews through descriptions of how respondents had to be creative with resources to support competence development. However, while this topic may be disproportionately prominent in this dataset, budgeting issues likely hold relevance to managers also in times of economic stability. Respondent 8 provided an example of long-term challenges with training in relation to resources:

Training takes time. It takes away from staff working hours, and we have long queues in some units. So, you must consider where to allocate resources. Even if we had unlimited money, we might still not send people to a lot of unnecessary training because the workforce is also needed. It's a very difficult balancing act.

##### 3.2.5.2 Adjusting schedules

Interviewees described scheduling night personnel during daytime to enable training participation and how to replace employees off to training, not rarely by stepping in themselves. Another type of management strategy in this subcategory was to designate time for learning and implementation. Respondent 11, described this in relation to implementation:

I think you must try to apply a higher degree of systematics than just approving a training and hoping that the person will come back and do this new thing. (…) The manager needs to be aware that performance will dip when someone is doing something new, and that you need to plan for it and even schedule it to a greater extent.

##### 3.2.5.3 Providing training materials

Interviewees described providing for materials needed for training and implementation, typically books—but also things like rooms, handouts and coffee during in-house training.

##### 3.2.5.4 Structuring new situations

Structuring new situations refers to managerial strategies that involve planning for changes to employees’ work environment or work processes. This was executed either through long-term changes of context (e.g., assign new tasks, locate the employee into a sub-specialist team, or provide supervision), or, by an isolated event (e.g., setting up an occasion to teach others what was learnt during training). Organizing opportunities to share knowledge was one of the most frequently employed strategies overall and was described in both positive and negative terms. Some saw knowledge-sharing opportunities as crucial since it meant letting all staff take part of the training content, hence rendered it mandatory, as respondent 6: “Just today, a nurse wanted to attend a training, and I said, ‘You can, but then you have to give an internal training right after.’” However, others described struggling with employees who dreaded and procrastinated the knowledge-sharing task, and some even declined to participate in training to avoid it.

## 4 Discussion

### 4.1 Main discussion

This study examined how mental health leaders describe the objectives of clinical staff training which they find meaningful, and the strategies they use to ensure those aims. The respondents viewed training as serving dual overarching purposes, relating to both care delivery and work environment—making it evident that the academic definitions of training outcomes (e.g., increased competence and patient improvement), do not fully reflect the broader image described by interviewees. This challenges the way educational efforts are normally evaluated in therapist training research, which tend to leave out the appraisal of goals relating to work environment and other objectives that are potentially important for the care providers. Despite that most literature on learning stress the need for clear training objectives, the respondents described that they rarely defined the specific goals of a specific training, a-priori. From a behavioral perspective, goal clarity functions as an antecedent that orients staff behavior and forms how training content and activities are selected, practiced, and reinforced (3). Furthermore, the importance of managers conveying clear goals prior to training is supported by implementation research, highlighting leadership's role in articulating goals and aligning them with organizational demands (34). The multitude of goals described by respondents in the current study—combined with limited strategies for communicating these goals to employees-may impair the effect of training by leaving staff uncertain about how they are expected to perform afterward. Therefore, these findings ought to be considered by leaders when planning training efforts, but also by both organizational management and researchers when evaluating the effects of training: Only training with an explicit goal of changes in clinical practice should be evaluated based on such outcomes.

While previous training literature mainly focuses on outcomes such as change in learners’ attitudes, knowledge and skills, there is some research supporting the interviewees’ notion that a prolific work environment can also be indirectly targeted through training. Previous studies has shown associations between access to competence development and staying on the job, and between employee's self-perceived competence and lower self-rated burnout among employees (35, 36). However, considering how costly staff training can be, there is also reason to investigate if some of those goals could be accomplished through other methods more closely related to the employees’ work environment.

Turning to the second research question addressing strategies and mechanisms of change, this study contributes with useful conceptualizations from an operant learning perspective. Overall, manager strategies represented a wide range of activities, across all predefined categories, before, during and after training suggesting that the framework provided a useful lens. Several of the reported strategies were used throughout the process—from selecting which staff to encourage to apply for training to the phase following training completion. For instance, *Antecedents* were used to prompt employees to apply for training, remind them to engage in training related behaviors, and by providing job-aids after training completion. Strategies relating to *Processes and resources* could be employed both during training by providing employees with time to study and study materials as well as structuring new situations where the employee is expected to use their new skills. Similarly, *Consequences* could be used to encourage staff to continue with their studies and to use their skills in their clinical practice and monitoring could be used both to check in on the staffś training progress and their work with their patients.

Despite describing a variety of strategies, managers also expressed concerns about how they performed the strategies. For instance, insufficient ongoing monitoring were described to lead to limited information about performance, making it difficult to evaluate both the training and to support their staff. Furthermore, participants described not being able to provide consequences in close temporal adjunction to performance, something that is needed for this strategy to be effective (37). At least two of the four strategies described within *Consequences*—offering financial incentives and providing feedback at annual performance reviews—may be too diluted and therefore function poorly as reinforcers of training outcomes. This also resonates with the implementation literature, suggesting that without systematic support from leaders, skills tend to not be effectively implemented (38, 39).

Within the category *Resources and processes*, the strategy *Structuring new situations* may indirectly fill some strategic gaps. Structuring assignment to sub-specialist teams or clinical supervision has the potential to create reinforcing contingencies, thus increasing the possibility of new skills to survive in the clinical context. For example, in supervision, the employee interacts with antecedents (prompts from the supervisor), monitoring (usually case recordings or self-assessment) and consequences (feedback) from the supervisor (40). Another type of structured situation—planned opportunities to report back to colleagues—seemingly have both advantages and drawbacks. From an operant learning perspective, it offers a chance to rehearse new knowledge, and informing others about the patients one can now treat may lead to more such referrals. However, it may also make training aversive and increase response effort, e.g., for employees who fear public speaking. As a one-time event, it is also less likely to have sustained impact on employee behavior.

While the interviewees as a collective described a broad repertoire of strategies, individual managers had their respective strong areas where they applied one or several strategies, and blind spots, i.e., domains with few or no strategies reported. They also described substantial obstacles such as lack of resources, competing training aims, and staff turnover. These barriers are well-documented in implementation science and pose challenges to mental health services (11). Furthermore, our findings indicate that training goals related to the work environment were rarely coupled with strategies to support their attainment—neither at the preparatory stage nor during follow up—thereby limiting feedback to managers regarding whether training is effective in this domain. If organizations are to uphold wellbeing- and work-environment-related training objectives, both leaders and training providers require greater knowledge of how training can be strategically designed and supported to achieve these outcomes. Addressing this gap should be a priority for future research.

### 4.2 Implications for practice

Our findings highlight that although the term ‘learning transfer’ can imply a simple process, like moving a set of skills from the classroom to the clinic, from an operant perspective, transfer is better understood as the planting of a seed: While training initiates behavior change, ongoing cultivation is essential for growth and sustainment. Hence, it is important that managers align training objectives with organizational needs and utilize impactful strategies. To achieve this, by drawing on this study's findings and operant learning principles (41), we propose five practical recommendations:

1. **Assess organizational needs** before initiating training, i.e., assess how the service is meeting its goals and identify specific challenges or gaps. These may include structural needs (e.g., inability to offer a specific intervention) or needs expressed by employees, like perceived skill deficits, low motivation, or emotional fatigue among staff. 2. **Verify that training is the appropriate solution** for identified challenges. For example, low adherence to clinical guidelines may stem from unclear expectations or impractical routines rather than a lack of competence. Work satisfaction or stress may be more effectively addressed through changes in workload or organizational support. With these things considered, in many cases, competence-building efforts remain a valid and valuable investment. 3. **Clarify and communicate intended outcomes**, including both clinical and work environment goals. 4. **Plan for transfer before training begins**, ensuring optimal conditions for practice, follow-up, and reinforcement. This can include allocating time and resources for training (e.g., protected time, course materials), preparing the workplace (e.g., providing manuals, checklists, treatment aids, patient referrals), and creating structures for follow-up and application (e.g., supervision, sub-teams, feedback systems functioning as reinforcing contingencies). 5. **Evaluate effects relative to the predefined organizational goals and needs**, using both objective indicators (e.g., service data, compliance rates) and subjective feedback (e.g., tailored post-training surveys or interviews with staff).

We recognize that managers in mental health services already carry significant responsibilities, and implementing structured approaches may be overwhelming. However, even low-effort strategies—such as brief, regular check-ins—can strengthen monitoring and provide timely reinforcement during the implementation process.

### 4.3 Strengths, limitations, and future directions

Because different contexts provide different opportunities for training and strategy use, a key strength of this study is the recruitment of managers from multiple levels across several Swedish regions. These findings are also likely to have relevance beyond Swedish psychiatric services, and future research should examine whether similar managerial perspectives appear in other health-care contexts and countries.

Furthermore, many managers in mental health services are trained in CBT, a therapeutic orientation largely informed by operant learning theory. As such, this approach may be familiar to its recipients, an important aspect when choosing a theoretical framework for research intended for implementation (42). Further, using a theory that has evidence-based applications in several fields, and that focuses on the function of strategies rather than providing a list of specific interventions that may work in one context but not another, strengthens the transferability of our method to other environments (43). Peer debriefings throughout the study further strengthened reflexivity and analytic rigor.

Limitations include reliance on managers’ accounts rather than observed behaviors or outcomes. Future research should examine how these strategies are experienced by staff, how they interact with contextual determinants, and whether operant-informed managerial interventions can improve the sustainment of training outcomes.

## 5 Conclusions

This study fills an important knowledge gap by highlighting the real-world perspective of leaders in mental health services. Clinical staff training was viewed by leaders as a versatile organizational tool and should be evaluated in relation to the organization's explicitly defined goals, which may extend beyond workforce competence and clinical impact. Applying operant learning theory clarifies why some strategies may succeed while others fall short. Understanding these mechanisms can help researchers, training providers, and service leaders strengthen the implementation and sustainment of clinical staff training initiatives within mental health organizations (44–47).

## Data Availability

The datasets presented in this article are not readily available because due to the sensitive and potentially identifiable nature of qualitative interview data, full transcripts cannot be shared without compromising participant anonymity. However, de-identified analytic materials (including coding trees and illustrative quotations) are available from the corresponding author upon reasonable request. Requests to access the datasets should be directed to emma.hogberg.ragnarsson@ki.se.

## Appendix 1

### Reflexivity and positionality statement

Four of the authors (EHR, SI, TL, LBN) are licensed psychologists with clinical experience and training in cognitive behavioral therapy; SI, a PhD, also specializes in organizational behavior management. EHR and SI work at a competence center providing training within mental health services, where TL is section head and research group leader. LBN holds a PhD and is head of department within psychiatry. CH is a psychiatrist, research group leader and professor in clinical psychology, and TS is a professor in Medical Education, without a background in the mental health services. Together, the team contributed with perspectives as clinicians, managers, trainees, trainers, and researchers.

We approached this study from a functional contextualist perspective (Biglan & Hayes, 1996; Gifford & Hayes, 1999), which emphasizes analyzing behavior in context for its pragmatic utility, rather than seeking underlying essences. Accordingly, we used an explorative approach to identify training objectives as described by managers. To analyze the function of the reported strategies, we applied operant learning theory, a framework consistent with functional contextualism (Biglan & Hayes, 1996).

## Appendix 2: Coding tree

**Table:**

Category		
Subcategory	Codes	Example quotes
Care delivery goals		
1. Day-to-day governance	• Keep and attract staff • Foster a common language • Foster similar work processes across the organization • Develop competencies difficult to recruit	“A generous approach to education and competence development increases our attractiveness as an employer. It makes it easier to recruit new psychologists and physicians.” (Respondent 15)
2. Clinical impact	• Produce patient benefits • Meet treatment processes and guidelines • Maintain safety for patients and clinicians	“First of all, training is about specific treatment methods we want to implement.” (Respondent 1)
Work environment goals		
3. Employee Wellbeing	• Support stress management • Reward staff • Let staff do what interest them	“It can be used a bit like a reward—that's not all that uncommon, I think. For employees we want to invest in and keep in the organization, the training can serve as an incentive.” (Respondent 3)
4. Connectedness among colleagues	• Facilitate prosocial activities • Support learning networks	“It's also a social event, and that connection between us who work together is really important”. (Respondent 12)
Bridging main categories		
5. Employee competence development	• Support staying updated and well-rehearsed • Handle low-performers • Foster self-monitoring • Stimulate at-work maturation • Support feelings of competence and growth	“The benefit is immense. We work with patients who have severe and sometimes lifelong diagnoses. We work with patients who have active suicidal thoughts, and sometimes suicide attempts. You need to have competence—and you need the confidence that competence provides—when engaging with these individuals.” (Respondent 5)
