# Supplementary material for: How leaders in mental health services shape workforce training outcomes: goals, actions, and mechanisms of change
Source: Front Health Serv. 2026 Apr 13;6:1784462. doi: 10.3389/frhs.2026.1784462 (PMC13111377; doi:10.3389/frhs.2026.1784462)
Supplement: Supplementary file 1 [file Supplementaryfile1.docx]

Supplementary Material 1: Interview guide

**INTERVIEW GUIDE**

**Welcome**

“Before we start recording, I will explain the purpose of the study and you will have the opportunity to ask questions.

Large resources are used for staff training. At the same time, we know that educational efforts not always lead to long-term changes in clinical practice. The purpose of this study is therefore to explore what managers in psychiatric services perceive as the values or objectives of clinical staff training. You are invited to participate in the study in your capacity as a manager or leader within psychiatric services. We expect the interview session to last no more than one hour.

Do you have any questions before we start recording?”

[START RECORDING]

Questions in bullet points, with the main question in plain style and optional follow-up questions/prompts in *italics*.

**Introduction**

“We will start by repeating the research information, and then you will answer yes or no to participate. It is the same information as when you signed up, but since some time has passed, we will repeat it”.

[Read information]

- If you want to participate in the interview by answering questions on the topics described above, please answer yes; otherwise, answer no.

“Now I will say something about the type of training this might involve. The study focuses on clinical continuing education. This can involve diagnostics, use of various scales, and, of course, psychological treatments of different conditions. We are interested in everything from shorter workshops, to longer courses such as basic or advanced psychotherapy training, and specialist training for various professional groups. Examples of training *not* included in our study are non-clinical trainings such as those related to fire safety, environment, and computer systems”.

- Do you feel like you understand what kind of training I mean?
- Do you have any examples of trainings of this type that your staff have participated in during the past year?

**Topic 1: Role in Training Issues**

“First, I want to know a bit about how you are involved in these matters”.

- What is your role in relation to this type of training issue?
- *How do you do that?*
- *When/how often?*
- *What is the purpose of those actions?*

**Topic 2: The Value of Training**

“The next section is about your perception of the benefits or objectives of employees participating in this type of training”.

- Can you tell me your thoughts about that?
- *Why is that important?*
- Are there any other objectives?

**Topic 3: Fulfilling goals/objectives/valued outcomes**

**“You mentioned these objectives: [list from previous question]”.**

- **Is there any way for you to know whether these objectives are fulfilled? Let’s start with [the first one, etc].**
- ***How/why not?***
- **What do you do (specific actions) to ensure or increase fulfillment? Let’s start with [the first one, etc].**
- ***How do you do that? Can you tell me more? How does that work?***

**Topic 4: Responsibility**

“The next section is about responsibility and the distribution of responsibility. If we consider three actors: those who conduct the training, those who attend the training, and you as the manager/representative of the organization”:

- How do you think the responsibility is distributed to ensure that the training fulfills its purpose, that is, achieves the values we have discussed?
- *How are these responsibilities taken? What actions?*
- *What are the purposes of those actions?*

**Topic 5: Challenges and wishes**

“Based on what you have described about how things are done today and how you work with continuing education and staff training; I would like to hear if there is anything you wished worked better?”

- What challenges do you meet in working with these training matters?
- How do you wish to work with training matters?
- *Why do you think that is important? How do you work with these difficulties or obstacles today? What is the purpose of those actions?*

**Conclusion**

“Now it’s time to wrap up this interview. I will just review my questions to see if there is anything else I need to add”.

- Is there anything else you would like to add regarding what we have discussed today?
- If you come to think of anything you want to add about what we talked about or how you experienced the interview, please feel free to get in touch.
- Thank you!

[END RECORDING]
