# Supplementary material for: How leaders in mental health services shape workforce training outcomes: goals, actions, and mechanisms of change
Source: Front Health Serv. 2026 Apr 13;6:1784462. doi: 10.3389/frhs.2026.1784462 (PMC13111377; doi:10.3389/frhs.2026.1784462)
Supplement: Supplementary file 2 [file Supplementaryfile2.docx]

Supplementary Material 2: COREQ checklist

| **Topic** | **Item No.** | **Guide Questions/Description** | **Reported on**  **Page No.** |
| --- | --- | --- | --- |
| **Domain 1: Research team**  **and reﬂexivity** | | | |
| *Personal characteristics* | | | |
| Interviewer/facilitator | 1 | Which author/s conducted the interview or focus group? | Materials and methods |
| Credentials | 2 | What were the researcher’s credentials? E.g. PhD, MD | Appendix 1 |
| Occupation | 3 | What was their occupation at the time of the study? | Appendix 1 |
| Gender | 4 | Was the researcher male or female? | Appendix 1 |
| Experience and training | 5 | What experience or training did the researcher have? | Appendix 1 |
| *Relationship with*  *participants* | | | |
| Relationship established | 6 | Was a relationship established prior to study commencement? | n/a |
| Participant knowledge of  the interviewer | 7 | What did the participants know about the researcher? e.g. personal  goals, reasons for doing the research | Supplementary material 1 |
|  |  |  |  |
| Interviewer characteristics | 8 | What characteristics were reported about the inter viewer/facilitator?  e.g. Bias, assumptions, reasons and interests in the research topic | Supplementary material 1 |
|  |  |  |  |
| **Domain 2: Study design** | | | |
| *Theoretical framework* | | | |
| Methodological orientation and Theory | 9 | What methodological orientation was stated to underpin the study? e.g. grounded theory, discourse analysis, ethnography, phenomenology,  content analysis | Materials & method, Appendix 1 |
|  |  |  |  |
| *Participant selection* | | | |
| Sampling | 10 | How were participants selected? e.g. purposive, convenience,  consecutive, snowball | Materials and methods |
| Method of approach | 11 | How were participants approached? e.g. face-to-face, telephone, mail,  email | Materials and methods |
|  |  |  |  |
| Sample size | 12 | How many participants were in the study? | Materials and methods |
| Non-participation | 13 | How many people refused to participate or dropped out? Reasons? |  |
| *Setting* | | | |
| Setting of data collection | 14 | Where was the data collected? e.g. home, clinic, workplace | Materials and methods |
| Presence of non-  participants | 15 | Was anyone else present besides the participants and researchers? | Materials and methods |
| Description of sample | 16 | What are the important characteristics of the sample? e.g. demographic  data, date | Materials and methods |
|  |  |  |  |
| *Data collection* | | | |
| Interview guide | 17 | Were questions, prompts, guides provided by the authors? Was it pilot  tested? | Materials and methods, Supplementary material 1 |
|  |  |  |  |
| Repeat interviews | 18 | Were repeat inter views carried out? If yes, how many? |  |
| Audio/visual recording | 19 | Did the research use audio or visual recording to collect the data? | Methods and materials |
| Field notes | 20 | Were ﬁeld notes made during and/or after the interview or focus group? |  |
| Duration | 21 | What was the duration of the inter views or focus group? | Methods and materials |
| Data saturation | 22 | Was data saturation discussed? | Methods and materials |
| Transcripts returned | 23 | Were transcripts returned to participants for comment and/or |  |
