# Supplementary material for: How leaders in mental health services shape workforce training outcomes: goals, actions, and mechanisms of change
Source: Front Health Serv. 2026 Apr 13;6:1784462. doi: 10.3389/frhs.2026.1784462 (PMC13111377; doi:10.3389/frhs.2026.1784462)
Supplement: Supplementary file 3 [file Supplementaryfile3.docx]

Reflexivity and positionality statement

Four of the authors (EHR, SI, TL, LBN) are licensed psychologists with clinical experience and training in cognitive behavioral therapy; SI, a PhD, also specializes in organizational behavior management. EHR and SI work at a competence center providing training within mental health services, where TL is section head and research group leader. LBN holds a PhD and is head of department within psychiatry. CH is a psychiatrist, research group leader and professor in clinical psychology, and TS is a professor in Medical Education, without a background in the mental health services. Together, the team contributed with perspectives as clinicians, managers, trainees, trainers, and researchers.

We approached this study from a functional contextualist perspective (Biglan & Hayes, 1996; Gifford & Hayes, 1999), which emphasizes analyzing behavior in context for its pragmatic utility, rather than seeking underlying essences. Accordingly, we used an explorative approach to identify training objectives as described by managers. To analyze the function of the reported strategies, we applied operant learning theory, a framework consistent with functional contextualism (Biglan & Hayes, 1996).
