# Supplementary material for: How leaders in mental health services shape workforce training outcomes: goals, actions, and mechanisms of change
Source: Front Health Serv. 2026 Apr 13;6:1784462. doi: 10.3389/frhs.2026.1784462 (PMC13111377; doi:10.3389/frhs.2026.1784462)
Supplement: Supplementary file 4 [file Supplementaryfile4.docx]

Appendix 2: Coding tree

| **Category**  Subcategory | Codes | Example quotes |
| --- | --- | --- |
| **Care Delivery Goals** |  |  |
| 1. Day-to-day governance | - Keep and attract staff - Foster a common language - Foster similar work processes across the organization - Develop competencies difficult to recruit | “A generous approach to education and competence development increases our attractiveness as an employer. It makes it easier to recruit new psychologists and physicians.” (Respondent 15) |
| 1. Clinical impact | - Produce patient benefits - Meet treatment processes and guidelines - Maintain safety for patients and clinicians | “First of all, training is about specific treatment methods we want to implement.”  (Respondent 1) |
| **Work Environment Goals** |  |  |
| 1. Employee Wellbeing | - Support stress management - Reward staff - Let staff do what interest them | “It can be used a bit like a reward – that’s not all that uncommon, I think. For employees we want to invest in and keep in the organization, the training can serve as an incentive.” (Respondent 3) |
| 1. Connectedness among colleagues | - Facilitate prosocial activities - Support learning networks | “It’s also a social event, and that connection between us who work together is really important”.  (Respondent 12) |
| **Bridging Main Categories** |  |  |
| 1. Employee competence development | - Support staying updated and well-rehearsed - Handle low-performers - Foster self-monitoring - Stimulate at-work maturation - Support feelings of competence and growth | “The benefit is immense. We work with patients who have severe and sometimes lifelong diagnoses. We work with patients who have active suicidal thoughts, and sometimes suicide attempts. You need to have competence – and you need the confidence that competence provides – when engaging with these individuals.”  (Respondent 5) |
